# In vitro and ex vivo gene expression profiling reveals differential kinetic response of HSPs and UPR genes is associated with PI resistance in multiple myeloma

**DOI:** 10.1038/s41408-020-00344-9

**Published:** 2020-07-28

**Authors:** Amit Kumar Mitra, Harish Kumar, Vijay Ramakrishnan, Li Chen, Linda Baughn, Shaji Kumar, S. Vincent Rajkumar, Brian G. Van Ness

**Affiliations:** 1 Department of Drug Discovery and Development, Harrison School of Pharmacy, Auburn University, Auburn, AL USA; 2 Department of Genetics, Cell Biology & Development, University of Minnesota, Minneapolis, MN USA; 3 Center for Pharmacogenomics and Single-Cell Omics initiative (AUPharmGx), Harrison School of Pharmacy, Auburn University, Auburn, AL USA; 4 Division of Hematology, Department of Internal Medicine, Mayo Clinic, Rochester, MN USA; 5 Division of Hematology/Oncology, School of Medicine, Indiana University, Indianapolis, IN USA; 6 Division of Laboratory Genetics, Department of Laboratory Medicine and Pathology, Mayo Clinic, Rochester, MN USA

**Keywords:** Myeloma, Cancer genomics, Cancer models

## Abstract

Extensive inter-individual variation in response to chemotherapy (sensitive vs resistant tumors) is a serious cause of concern in the treatment of multiple myeloma (MM). In this study, we used human myeloma cell lines (HMCLs), and patient-derived CD138+ cells to compare kinetic changes in gene expression patterns between innate proteasome inhibitor (PI)-sensitive and PI-resistant HMCLs following test dosing with the second-generation PI Ixazomib. We found 1553 genes that changed significantly post treatment in PI-sensitive HMCLs compared with only seven in PI-resistant HMCLs (*p* < 0.05). Genes that were uniquely regulated in PI-resistant lines were RICTOR (activated), HNF4A, miR-16-5p (activated), MYCN (inhibited), and MYC (inhibited). Ingenuity pathway analysis (IPA) using top kinetic response genes identified the proteasome ubiquitination pathway (PUP), and nuclear factor erythroid 2-related factor 2 (NRF2)-mediated oxidative stress response as top canonical pathways in Ix-sensitive cell lines and patient-derived cells, whereas EIF2 signaling and mTOR signaling pathways were unique to PI resistance. Further, 10 genes were common between our in vitro and ex vivo post-treatment kinetic PI response profiles and Shaughnessy’s GEP80-postBz gene expression signature, including the high-risk PUP gene PSMD4. Notably, we found that heat shock proteins and PUP pathway genes showed significant higher upregulation in Ix-sensitive lines compared with the fold-change in Ix-resistant myelomas.

## Introduction

Multiple myeloma (MM) remains an incurable disease with 5-year survival rate of 48.5% (NCI-SEER Cancer statistics)^1^. Extensive inter-individual variation in response to chemotherapy is a serious cause of concern in the treatment of myeloma^2,3^. Not all patients respond equally well to treatment and those who do often develop resistance over the course of treatment. Drug resistance may therefore be categorized into: (1) innate resistance already present in drug-naive patients who never respond to treatment, or (2) emerging/acquired resistance where a patient’s tumor ultimately undergoes relapse or “acquires” the ability to resist therapy in course of treatment despite good response to initial treatment^3,4^. Proteasome inhibitors (PIs) are standard-of-care chemotherapeutic agents for myeloma that impede tumor metastasis and angiogenesis by accelerating unfolded protein response (UPR) and by interfering with the nuclear factor-κB-enabled regulation of cell adhesion-mediated drug resistance^3,5,6^.

Alterations in gene expression levels have been shown to be associated with response to cancer drugs, including PIs in myeloma^7,8^. In our previous study, we used a panel of 50 human myeloma cell lines (HMCLs) representing the broad spectrum of biological and genetic heterogeneity of myeloma, and generated in vitro drug response profile for the four PIs: bortezomib (velcade/Btz), carfilzomib (kyprolis/Cfz), oprozomib (Opz), and ixazomib (ninlaro/Ix) as single agents. As PI sensitivity in these HMCLs were highly correlated (given a common target), this suggested that any of these four PIs could be used as surrogates in models to understand common PI resistance. Further, we used machine learning-based computational approaches to derive a gene expression profiling (GEP) signature predictive of baseline PI response in myeloma^9^.

Here, we were interested in understanding how gene expression patterns may be differentially influenced in sensitive vs resistant myelomas following exposure to PIs (kinetic PI response). Therefore, to characterize differences in kinetic PI response, we compared transcriptome profiles of HMCLs in vitro as well as patient primary tumor cells ex vivo representative of highly PI-sensitive and PI-resistant myelomas between baseline and post treatment following test dosing with the model PI drug ixazomib (Ix)^3^. Our results provide significant insights into the wide heterogeneity by identifying key differences in PI-specific kinetic responses.

## Materials and methods

### Drugs

Ix and Bz was procured from Takeda (Takeda Pharmaceuticals Inc., Deerfield, IL, USA). Cfz and Opz were obtained from Amgen (Thousand Oaks, CA, USA). Drugs were dissolved in dimethyl sulfoxide and stored at −20 °C.

### Antibodies

HSP40 (DNAJB1), HSPA1b (HSP70), HSP90alpla antibodies were purchased from Enzo Life Sciences (Farmingdale, NY, USA). Monoclonal anti-β-actin-peroxidase antibody produced in mouse was from Sigma-Aldrich (St Louis, MO, USA). Goat anti-Mouse IgG (H + L) Secondary antibody (horseradish peroxidase (HRP) conjugated) was obtained from Thermo Fisher Scientific (Waltham, MA, USA).

### Cell lines

Twelve (12) myeloma cell lines—established, characterized, and authenticated by our collaborators at The Translational Genomics Research Institute (TGen) and NIH representing variabilities in PI-response in myeloma patients were selected from our HMCL panel described previously^10^.

### Patient samples

CD138-selected plasma cells were derived from myeloma patients enrolled in a phase-2 Ix clinical trial at Mayo Clinic, Minnesota (Mayo-Ix; NCT01415882). These were relapsed myeloma patients had less than six cycles of prior treatment with a PI-based regimen and were not refractory to the PI drug bortezomib (Bz)^9^.

### In vitro chemosensitivity assays

Cells (HMCLs and patient-derived primary cells) were counted using countess automated cell counter (Invitrogen, Carlsbad, CA, USA) and seeded in 96-well plates at a concentration of 4 × 10^5^ cells per ml. Twenty-four (24 h) hours later, Ix, representative PI drug, was added as a single agent in increasing concentrations. Forty-eight (48 h) hours post Ix treatment, CellTiter-Glo luminescent cell viability assay (Promega, Madison, WI, USA) was used to perform cell viability assays according to manufacturer’s instructions using Synergy 2 Microplate Reader (BioTek, Winooski, VT, USA). Survival curves were generated, percent survival values were calculated at each drug concentration and normalized to untreated controls. Half-maximal inhibitory concentration (IC_50_) values were computed by nonlinear regression using sigmoidal dose–response equation (variable slope). Area under survival curve (AUSC) was calculated using trapezoidal rule^9^.

### Cell plating for kinetic Ix treatment and RNA-sequencing

HMCLs and patient-derived primary myeloma tumor cells were plated at a density of 4 × 10^5^ cells per mL. In all, 15 nm (IC_50_ of the most sensitive HMCL) and 40 nm (median IC_50_ of patient cells) of Ix were added to HMCLs and PMCs, respectively, after 24 hours of incubation. Cells were collected 24 hours post-treatment as baseline (0 nm/untreated) and post-treatment (treated). High-quality RNA was extracted from untreated and treated myeloma cells using QIAshredder and RNeasy kit (Qiagen). RNA concentration and integrity were assessed using the Nanodrop-8000 and Agilent 2100 Bioanalyzer and stored at −80 °C. RNA integrity number/RIN threshold of eight was used for RNA-seq analysis. RNA-seq libraries were constructed using Illumina TruSeq RNA sample Preparation kit v2. Libraries were then size selected to generate inserts of ~200 bp. RNA sequencing was performed on llumina’s HiSeq 2000 next-generation high-throughput sequencing system using 50 bp paired-end protocol with depth of >20million reads per sample. Average quality scores were thoroughly above Q30 for all libraries in both R1 and R2.

### RNA-Seq data processing

RNA-seq data were normalized and fragments per kilobase million values were used in further analysis using Partek Genomics Suite and Galaxy data analysis software, an open source, web-based platform that provides tools necessary to create and execute RNA-seq analysis. In brief, RNA-seq data analysis pipeline was developed using Galaxy software workflow. Quality control (QC) check on the RNA-seq raw reads was performed using FastQC tool followed by read trimming to remove base positions that have a low median (or bottom quartile) score. Tophat2 tool mapped processed RNA-seq reads to the hg19 human genome build. Picard’s CollectInsertSizeMetrics tool was applied on the initial tophat2 run to obtain estimated insert sizes, which was then used to calculate mean inner distance between mate pairs (mean = estimated_insert-size−2×read_length). Tophat2 was re-run using corrected mean value and Cufflinks tool was to assemble the reads into transcripts.

### Bioinformatics and statistical analysis

Gene expression data were pre-processed using Galaxy platform and filtered using the following criteria: genes with mean FPKM < 1 were removed. GEP data were analyzed further using a combination of Galaxy and Partek Genomics Suite v7.0 to perform differential expression testing to identify GEP signatures of kinetic PI-response. Mean fold-change >|1| and *p* < 0.05 was considered as threshold for reporting significant differential gene expression.

All statistical analyses were performed using R for statistical computing and graphics, version 3.4.2, and GraphPad Prism version 7.0. All tests were two-sided and *p* < 0.05 was considered statistically significant.

Differentially expressed (DE) gene analysis was performed between two groups of gene expression data sets (e.g., treated vs untreated). For HMCL data, we first detected DE genes for: (1) all the 12 HMCLs combined between treated (15 nm) and untreated groups; (2) six sensitive HMCLs and six resistant HMCLs between treated (15 nm) and untreated groups; separately.

Similarly, for patient data, we also performed DE analysis for: (1) eight patients and (2), separately, among four sensitive patients and four resistant patients between treated (40 nm) and untreated groups. Owing to the small sample size, we use limma, an empirical Bayesian method, to detect the DE genes. The advantage of limma compared with traditional *t* test is that limma provides a moderated *t* test statistic by a shrinking the variance statistics, therefore, improves the statistical power. Thus, we obtained the *p* value for each DE gene and further provided FDR based on the *p* value using Benjamini–Hochberg procedure. Genes with *P* < 0.05 are considered as DE genes.

### Ingenuity pathway analysis

Ingenuity pathway analysis (IPA) software (QIAGEN) was be used to identify the most significantly affected molecular pathways predicted to be activated or inhibited in response to secondary drugs and in stem-cell based PI resistance modeled on list of significantly differentially regulated genes. IPA is a web-based software application that integrates and interprets the data derived from differential mRNA expression analysis to: (i) identify the most significantly affected canonical pathways predicted to be activated or inhibited; (ii) predict upstream regulator molecules like microRNA and transcription factors, which may be causing the observed gene expression changes; (iii) analyze downstream effects and biological processes that are increased or decreased; (iv) predict causal networks; and (v) perform predictive toxicology analysis using toxicogenomics approaches (IPA-Tox)^11^.

### Western blotting

Myeloma cells were cultured with (treated) or without (untreated) Ix (15 nm), harvested, washed, and lysed using radioimmunoprecipitation assay lysis buffer containing 50 mm Tris-HCl, pH 7.5, 150 mm NaCl, 1% NP40, 5 mm EDTA, 1 mm DTT, phosphatase, and protease inhibitors cocktail (Sigma) and incubated on ice for 15 min. Samples were then centrifuged at 14,000 rpm at 4 °C for 30 mins. The supernatant was then aspirated and quantified using Pierce BCA Protein Assay Kit (Thermo Scientific). Samples were solubilized in sodium dodecyl sulfate polyacrylamide gel electrophoresis sample buffer, and equal amounts of protein were loaded per lane of 10% SDS–PAGE and transferred onto polyvinylidene difluoride membranes (Millipore; Billerica, MA). Membranes were blocked in tris-buffered saline (TBS) with SuperBlock blocking buffer (Thermo Fisher). Membranes were incubated with primary antibodies for HSP40 (DNAJB1), HSPA1b (HSP70), and HSP90alpla and secondary antibodies in TBS with 0.2% Tween 20 and 2.5% bovine serum albumin. Immunoreactivity was detected by Chemiluminescent HRP substrate (Bio-Rad) and the exposed image was captured using a ChemiDoc MP Imaging System (Bio-Rad). Densitometry analysis was performed (in triplicates) using Image J software.

## Results

### In vitro and ex vivo cell survival assays and patient outcome

**Fig. 1:**
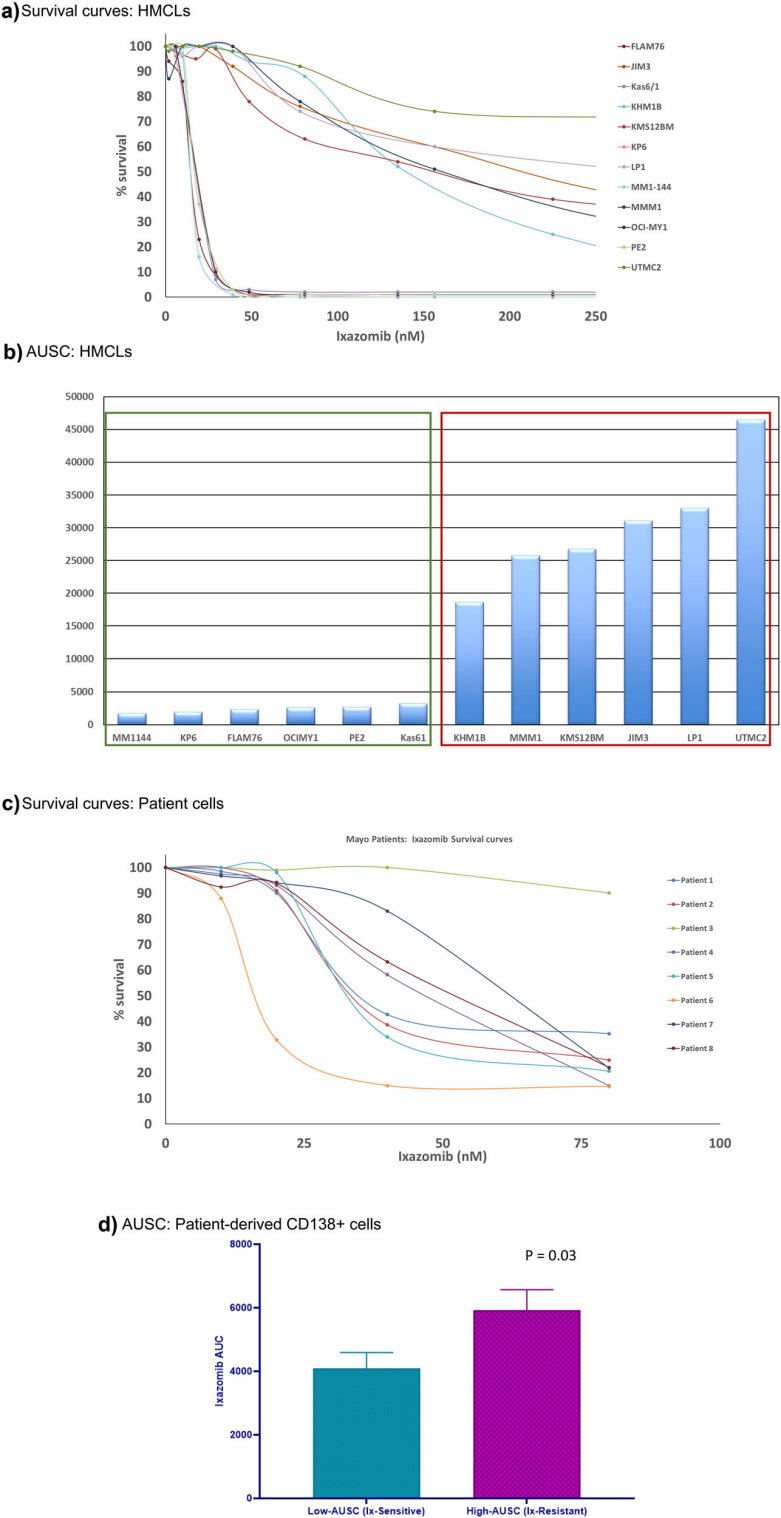
In vitro and ex vivo Ixazomib response in human myeloma cell lines and myeloma patient-derived cells. Survival curves show percent survival compared with untreated control at increasing concentrations of Ixazomib. Bar graphs represent the AUSC values for all cells treated with the proteasome inhibitor drug Ixazomib. **a** Survival curves: HMCLs; **b** AUSC: HMCLs; **c** survival curves: patient cells; **d** AUSC: patient-derived CD138+ cells.

Response to Ix in HMCLs and patient-derived human myeloma cells are provided in Fig. 1a, b. As some of the test cells (PMCs and HMCLs) did not achieve IC_50_, we used AUSC values to assess relative sensitivity to Ix and performed binary grouping into Ix-sensitive and Ix-resistant subgroups, as highlighted in the figure. In vitro response to Ix in eight primary myeloma patient PCs is shown in Fig. 1c, d. Patient outcome data in terms of response to PI-containing chemotherapy regimen showed all the patients in the low Ix AUC category (in vitro PI-sensitive) presented with some form of response (CR/PR/VGPR), whereas only one patient in the PI-resistant (high in vitro cytotoxicity) category showed complete response (Fig. S1a). Median progression-free survival values were 371 days for the PI-resistant patients and 858.5 for the Ix-sensitive patients (Hazards ratio >1.5) (Fig. S1b).

### Differential kinetic GEP of HMCLs

**Fig. 2:**
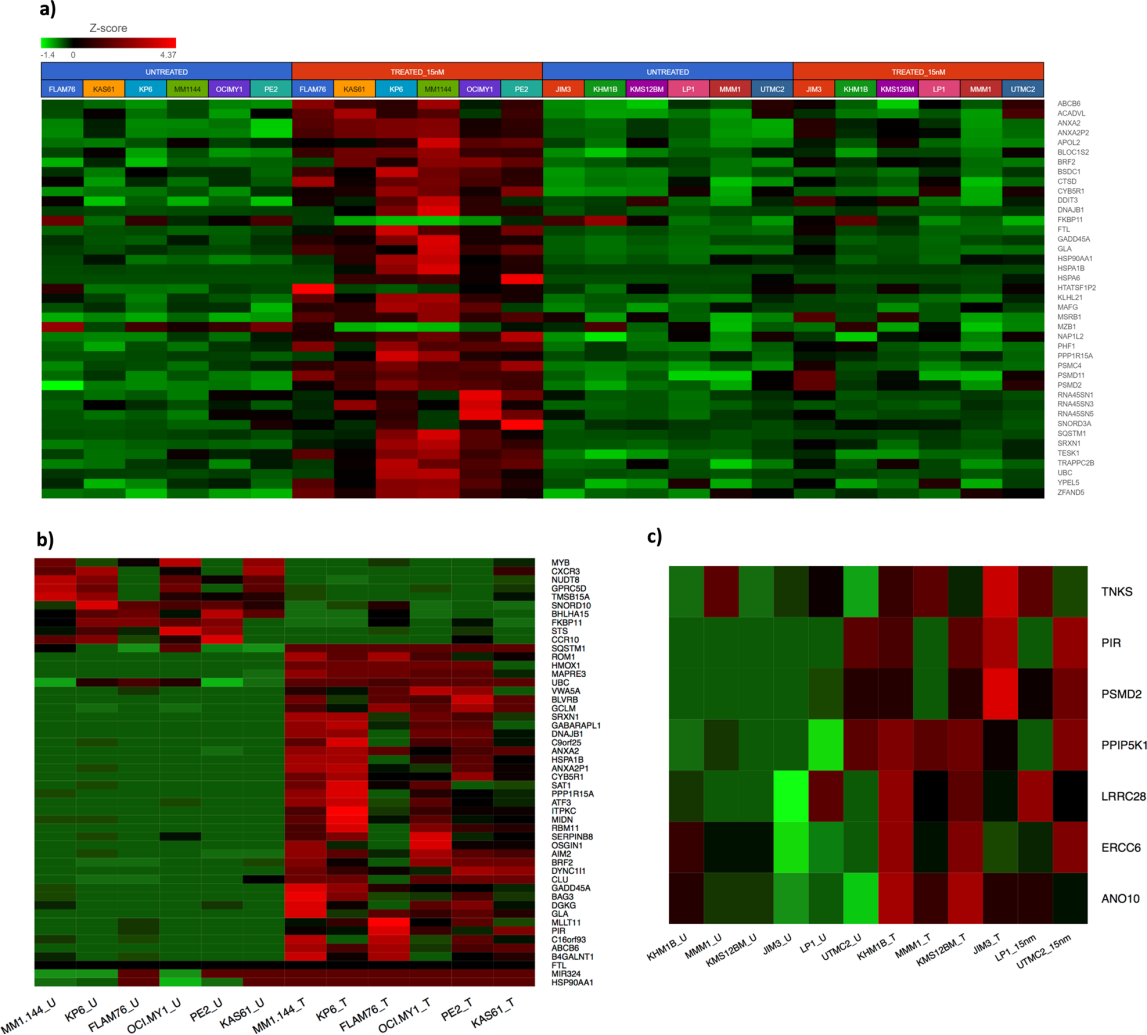
Heatmaps representing kinetic gene expression profiles of human myeloma cell lines. **a** Unsupervised hierarchical clustering (HC) analysis showing differential gene expression of top genes in 12 myeloma cell lines (six sensitive, six resistant) that showed significant de-regulation (|fold-change| > 1.5; *p* < 0.05) in response to Ixazomib treatment, 24 h following drug exposure (Ixazomib-15nm). Columns represent cell lines and rows represent genes. Prior to hierarchical clustering, gene expression values were filtered (samples with mean FPKM < 1 or max FPKM < 1 were removed) and *z* score normalized. Columns are ordered by Ix-response followed by Ix-treatment. **b** Heat map of Top 50 genes that changed significantly (*p* < 0.05) between baseline and post-treatment Ix-sensitive myeloma cell lines (*n* = 6). Columns are ordered by Ix-treatment. **c** Heat map of top genes (*p* < 0.05) that changed significantly between baseline and post-treatment Ix-resistant myeloma cell lines (*n* = 6). Columns are ordered by Ix-treatment.

In order to identify kinetic changes in gene expression patterns common to all the HMCLs we first performed a combined analysis of all 12 HMCLs (Ix-sensitive + Ix-resistant) considered together between baseline and 24 h post Ix treatment of 15 nm. A total of 583 DE genes displayed *p* value > 0.05 (|fold-change | >1; *p* < 0.05) and 304 genes at |fold-change | >1.5 (*p* < 0.05). Figure 2a shows a heatmap of top genes in 12 myeloma HMCLs (six sensitive, six resistant) that showed significant change in expression of in response to Ix treatment, 24 hours following drug exposure (Ix-15 nm). IPA analysis of the 583 genes identified the proteasome ubiquitination pathway (PUP) (*P* = 6.19E-25; 45 genes) and nuclear factor erythroid 2-related factor 2 (NRF2)-mediated oxidative stress response pathway (*P* = 6.93E-06) as the top canonical pathways that respond to the PI treatment.

However, we observed that the GEP data were highly influenced by the DE genes in sensitive HMCLs. Therefore, to understand major differences in differential kinetic gene expression patterns between Ix-sensitive and Ix-resistant lines, analysis of untreated baseline and post 15 nm Ix treatment was performed on the six most Ix-sensitive and six most Ix-resistant HMCLs, separately (Fig. S2).

When Ix-sensitive lines were considered alone, 1553 genes changed significantly post-treatment with any change (|fold-change| > 1; *p* < 0.05). In total, 1167 genes at |fold-change| >1.5 (*p* < 0.05). The top 50 most highly regulated genes for each condition are listed in Table S1 a–d. A heatmap for the top 50 genes in Ix-sensitive lines is provided in Fig. 2b.

**Fig. 3:**
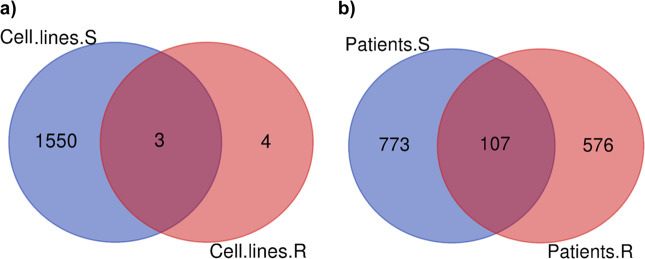
Venn diagrams illustrating common and unique kinetic DE genes. Venn diagrams showing comparison of the kinetic response profiles between Ix-sensitive and Ix-resistant **a** HMCLs (*p* < 0.05); **b** Patients cells (*p* < 0.05) (baseline vs 40 nm).

On the other hand, kinetic response profiling in Ix-resistant HMCLs revealed at *p* < 0.05, only seven genes showed any significant change (|fold-change| >1; *p* < 0.05) (Fig. 2c). Comparison of the distinct kinetic response profiles of the Ix-sensitive and Ix-resistant HMCLs revealed three common DE genes (PIR, TNKS, PSMD2) that changed significantly in both the lists at *p* < 0.05 as shown in the venn diagram in Fig. 3a. Four genes were unique to the Ix-resistant HMCLs only—ANO10, ERCC6, LRRC28, PPIP5K1.

IPA analysis of the most DE genes in Ix-sensitive and Ix-resistant HMCLs in response to Ix-test dosing showed that the top canonical pathway was the PUP (*p* = 2.64E-23 and 1.96E-08 for sensitive and resistant HMCLs, respectively) (Fig. S3). IPA-Tox analysis in Ix-sensitive HMCLs revealed 33 genes overlapped with the top pathway NRF2-mediated oxidative stress response (*P* = 6.26E-05), whereas nine genes overlapped with oxidative stress (*P* = 9.51E-03).

A comparison of the major PUP and stress response pathway genes between the Ix-sensitive and Ix-resistant lists revealed that the three of the top four genes that showed significantly different kinetic response were DNAJB1 (HSP40), HSPA1B (HSP70), and HSP90AA1 with sensitive to Resistant fold-change ratios of 31.6, 6.3, and 4.4, respectively. Furthermore, all the seven PUP genes (BIRC6, PSMA1, PSMB3, PSMB5, PSMC4, PSMC6, PSMD2) that were common between the two lists that showed >1.5-fold higher upregulation in Ix-sensitive lines (Table 1).

**Table 1:** Comparison of kinetic expression of heat shock proteins and protein ubiquitination pathway genes between Ix-sensitive and Ix-resistant human myeloma cell lines (|(fold-change _Ix-sensitive_)| >1.5; *p* < 0.05).

	Fold-change sensitive	*P* value sensitive	Fold-change resistant	*P* value resistant	Ratio of fold-change (sensitive/resistant)
HSPA1B	41.511	3.85E-02	1.314	2.15E-01	31.593
DNAJB1	6.924	2.40E-02	1.096	6.68E-01	6.315
UBC	5.563	2.17E-02	1.076	5.03E-01	5.171
HSP90AA1	4.810	1.70E-02	1.085	6.86E-01	4.432
DNAJB4	3.483	2.56E-02	1.125	8.12E-01	3.097
DNAJB2	2.661	2.00E-03	1.093	8.19E-01	2.434
PSMD12	2.667	2.19E-06	1.156	4.77E-01	2.307
PSMC6	2.910	3.06E-03	1.289	7.12E-02	2.258
DNAJC18	2.261	3.60E-02	1.023	9.32E-01	2.210
PSMC4	3.360	4.12E-05	1.544	9.75E-02	2.177
PSMC1	2.940	6.60E-03	1.382	1.48E-01	2.127
SKP2	−2.143	2.80E-04	−1.039	7.99E-01	2.063
PSMD6	2.402	5.73E-05	1.186	3.01E-01	2.026
TRAP1	−2.195	1.32E-02	−1.093	6.80E-01	2.008
ZBTB12	−2.379	2.59E-02	−1.197	1.07E-01	1.987
PSMB7	2.551	1.65E-04	1.363	1.51E-01	1.872
TRAF6	1.858	2.08E-02	1.002	9.89E-01	1.855
HSP90AB1	1.990	2.79E-03	1.073	7.23E-01	1.854
USP14	2.116	7.06E-04	1.150	4.19E-01	1.840
PSMD14	2.500	3.89E-06	1.359	1.24E-01	1.839
PSMD1	2.391	1.06E-03	1.324	1.26E-01	1.806
PSMD11	2.581	8.33E-05	1.438	2.48E-01	1.795
BRCA1	−1.853	1.21E-03	−1.053	8.66E-01	1.760
PSMD2	2.583	3.35E-05	1.482	4.98E-02	1.743
UBE2T	−1.783	2.64E-02	−1.028	9.40E-01	1.734
PSMB2	2.392	3.31E-07	1.380	1.24E-01	1.733
PSMD13	2.213	3.16E-03	1.290	1.20E-01	1.716
ANAPC10	1.683	6.26E-03	1.010	9.34E-01	1.666
USP5	2.085	2.14E-05	1.284	4.25E-01	1.624
PSMC5	2.213	9.17E-05	1.368	2.75E-01	1.618
PSMA6	1.902	2.45E-02	1.186	3.90E-01	1.604
PSMB5	2.052	3.72E-03	1.280	6.89E-02	1.603
PSMD3	2.202	1.14E-05	1.383	1.73E-01	1.592
PSMA1	2.101	7.45E-04	1.335	9.98E-02	1.574
UBR2	1.753	5.27E-05	1.157	5.53E-01	1.515
PSMB3	1.980	6.22E-04	1.308	9.08E-02	1.514
USP47	1.711	1.31E-02	1.131	5.64E-01	1.514
PSMD8	1.763	1.44E-02	1.170	2.72E-01	1.507
ANAPC1	−1.566	7.39E-03	−1.042	8.77E-01	1.503
DNAJC19	−1.581	2.74E-02	−1.058	7.75E-01	1.495
DNAJC25	−1.615	1.97E-03	−1.081	4.75E-01	1.494
PSMD4	2.144	3.46E-02	1.436	3.05E-01	1.493
USP30	1.824	7.14E-03	1.237	2.36E-01	1.475
PSMC2	1.869	3.54E-04	1.267	2.17E-01	1.475
PSMB4	2.041	1.84E-02	1.389	1.36E-01	1.469
PSME1	−1.559	4.32E-02	−1.066	7.74E-01	1.462
SKP1	1.717	3.14E-02	1.176	5.28E-01	1.461
PSMB6	2.030	6.49E-03	1.390	2.61E-01	1.460
PSMA4	1.762	1.23E-03	1.221	2.05E-01	1.443
PSMC3	1.923	4.86E-04	1.343	2.75E-01	1.432
UBE2R2	1.505	1.23E-02	1.062	7.74E-01	1.417
PSMA2	1.590	2.62E-04	1.137	5.62E-01	1.398
UBR1	1.546	1.59E-02	1.141	2.43E-01	1.355
PSMA7	1.605	1.62E-02	1.210	1.62E-01	1.327
UBE4B	1.525	1.94E-03	1.160	3.54E-01	1.315
BIRC6	1.506	2.41E-02	1.162	9.87E-02	1.296
UBE2S	−1.556	2.20E-02	1.044	8.72E-01	−1.490

### Differential kinetic GEP of patient-derived myeloma cells

**Fig. 4:**
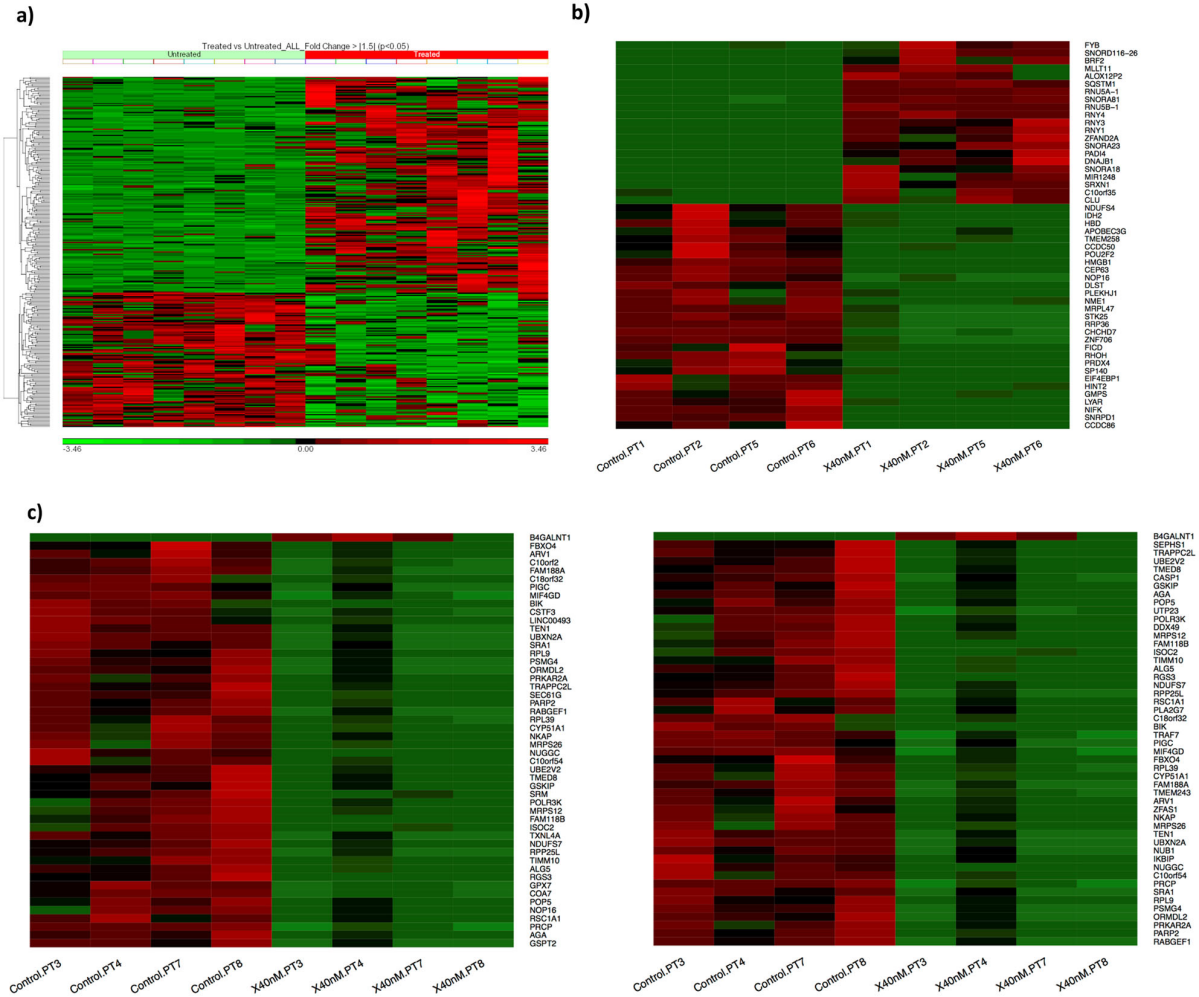
Heatmaps representing kinetic gene expression profiles of patient-derived primary myeloma cells. **a** Unsupervised hierarchical clustering (HC) analysis showing differential gene expression of 296 genes in patient-derived myeloma cells (*n* = 8) that showed significant de-regulation (|fold-change| > 1.5; *p* < 0.05) in response to Ixazomib treatment, 24hours following drug exposure. Color indicates fold-change between baseline and 40nm Ix treatment. Columns represent cell lines and rows represent genes. Prior to hierarchical clustering, gene expression values were *z* score normalized. **b** Heat map of Top 50 genes that changed significantly (*p* < 0.05) between baseline and post-treatment Ix-sensitive myeloma patient cells (*n* = 4); color indicates fold-change between baseline and 40nm Ix treatment. **c** Heat map of top genes that changed significantly (*p* < 0.01) between baseline and post-treatment Ix-resistant myeloma patient-derived cells (*n* = 4). Color indicates fold-change between baseline and 40 nm Ix treatment.

We subsequently isolated CD138+ cells from myeloma patients and tested the in vitro response to Ix. Even in this small sampling, we noted a diverse response (Fig. 1c, d). An overall analysis of differential gene expression between all the untreated and Ix-treated patient-derived myelomas revealed the following: 1284 genes had a |fold-change| >1 at *p* < 0.05. As shown in Fig. 4a, 296 of these genes showed highly significant altered expression with |fold-change| >1.5 and *p* < 0.05). When the top four most Ix-sensitive patient-derived cells, based on corresponding AUSC values, were considered separately, 880 genes were shown to change significantly between untreated vs treated conditions |fold-change| >1 (*p* < 0.05). The top 50 genes are shown in the heatmap (Fig. 4b). IPA analysis revealed the following as the top canonical pathways for the Ix-sensitive patients: PUP (*P* = 1.46E-14; overlap = 14.9% or 40/269); mitochondrial dysfunction (*P* = 9.34E-07; overlap = 11.7% or 22/188), NRF2-mediated oxidative stress response (*P* = 2.43E-06; overlap = 11.1% or 22/199); UPR (*P* = 2.37E-05; 17.9% or 10/56); and vascular endothelial growth factor signaling (*P* = 5.61E-05; 12.2% or 14/115). Notably, XBP1 inhibition (*Z* score = −4.297) and NFE2L2 activation (*Z* score = 3.095) were predicted as top upstream regulators of these treatment-influenced differentially regulated genes with *P* = 4.77E-12 and 2.92E-11, respectively.

Furthermore, 210 of these genes were also found differentially regulated in Ix-sensitive HMCLs. 23 of these genes belong to the PUP (*P* = 3.51E-16) including the following proteasome subunit genes PSMA4, PSMA6, PSMB2, PSMB4, PSMB5, PSMC2, PSMC3, PSMC4, PSMC6, PSMD1, PSMD11, PSMD2, PSMD3, PSMD4, PSMD6, PSME4, whereas 12 genes belong to the NRF2-mediated oxidative stress response (*P* = 2.47E-07).

On the other hand, kinetic gene expression analysis of Ix-resistant PMCs with lower four AUSCs demonstrated 683 genes with significant |fold-change| (>1) between baseline vs post-treatment with significant *p* value (*p* < 0.05). Heatmap for the top 50 most differentially regulated genes is represented in Fig. 4c. The gene lists are provided in Table S1c (Ix-sensitive) and S1d (Ix-resistant).

When compared, 107 kinetic genes were common between the Ix-sensitive and Ix-resistant patients, whereas 576 genes were unique in Ix-resistant PMCs only, as demonstrated in the venn diagram (Fig. 3b).

Supplementary figure S4 shows venn diagrams obtained from the kinetic response profiling of all the subgroups (HMCLs and patients; PI- sensitive and resistant) and depicting the comparison of all the significant gene lists (*p* < 0.05).

IPA analysis of these 576 PI kinetic genes unique to the Ix-resistant patients showed EIF2 Signaling (*P* = 3.04E-10; overlap = 10.9% or 25/230), regulation of eIF4 and p70S6K signaling (*P* = 5.48E-07; overlap = 10.2% or 17/167) and mTOR signaling (*P* = 3.84E-06; overlap = 8.5% or 18/213) as top canonical pathways. The top IPA-Tox prediction for Ix-resistant patients were RAR activation (*P* = 6.03E-03; overlap = 5.8% or 11/190) and mitochondrial dysfunction (*P* = 9.77E-03; overlap = 5.7% or 10/176). The top predicted upstream molecules that may be causing the observed gene expression changes were RICTOR activation (*Z* score = 4.707; *P* = 4.07E-09) and miR-16-5p (*Z* score = 2.903; *P* = 4.35E-06); whereas MYCN (*Z* score = −3.017; *P* = 1.03E-05), MYC (*Z* score = −3.632; *P* = 1.49E-05) and EIF4E (*Z* score = −2.358; *P* = 2.08E-04) were predicted to be inhibited.

### Ix induced higher kinetic upregulation of HSPs in sensitive cells compared to resistant cells

**Fig. 5:**
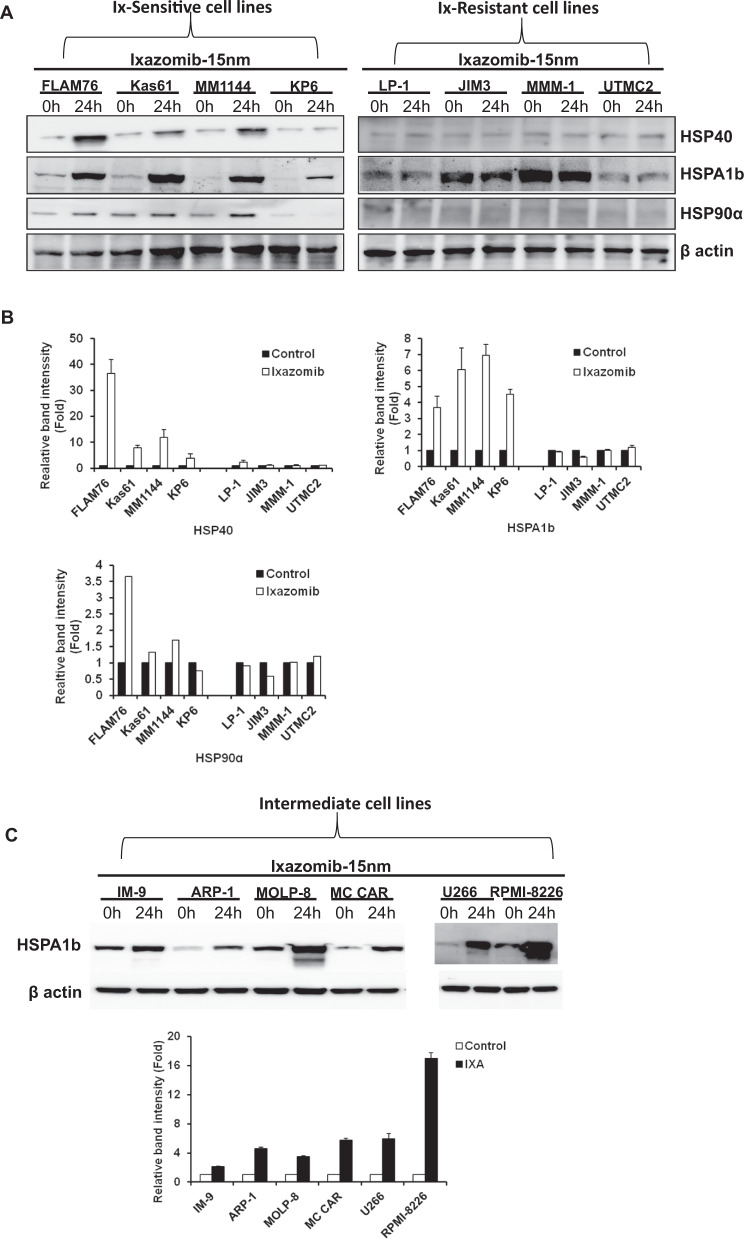
Effect of Ixazomib on heat shock protein (HSP’s) levels in multiple myeloma cell lines. Ix-sensitive cell lines (FLAM76, Kas6/1, MM1-144, KP6) and Ix-resistant cell lines (LP1, JIM3, MMM1, UTMC2) were treated with 15 nm of Ixazomib for 24 h followed by protein harvest and western blot analysis. **a** Representative western blot images. **b** Representative protein densitometry analysis. **c** Ix-intermediate response cell lines (IM-9, ARP-1, MOLP-8, MC CAR, U266, and RPMI-8226).

As our in silico study showed that Ix-sensitive cells have significantly elevated of HSP70b levels post treatment compared with Ix-resistant cells, we evaluated heat shock protein (HSP) expression in a panel of PI-sensitive and -resistant cells at protein level to validate further the genomic data. Following treatment with Ix for 24 h at 15 nm concentration, Ix-sensitive cells showed higher expression of HSP40 (DNAJB1), HSPA1b (HSP70), HSP90alpla compared with the increase in expression in Ix-resistant cells (Fig. 5). Thus, western blotting confirmed fold-change differences in kinetic response between sensitive vs resistant lines.

## Discussion

Extensive inter-individual variation in response to chemotherapy is a serious cause of concern in the treatment of MM. Using a panel of HMCLs, we have earlier presented a GEP signature that could not only distinguish good and poor PI-response in the HMCL panel, but was also successful in stratifying exceptional response to PI-based chemotherapy in myeloma clinical data sets^9^.

Although a recent study used CRISPR-based screening approaches to identify genes associated with PI response in myeloma, no study so far has combined in vitro and ex vivo pre- and post-treatment gene expression profiles to compare PI treatment-induced gene signatures between sensitive and resistant myelomas. In this study, we used six most PI-sensitive and six most innate PI-resistant myeloma lines from our HMCL panel above and identified in vitro the kinetic changes in gene expression patterns following Ix test dosing. Subsequently, we also performed an ex vivo analysis of kinetic gene expression changes between untreated and treated myeloma tumor cells derived from ongoing Ix-containing clinical trials. Interestingly, our ex vivo Ix-sensitivity data corroborated with patient PI response from follow-up data. Our analysis identified sets of genes that changed significantly post-treatment in Ix-sensitive and comparatively Ix-resistant myelomas with significant similarities and some notable differences. These therefore represent distinct signatures of kinetic response to Ix in sensitive and innate resistant myeloma and highlight major differences in pathway regulation associated with differential treatment responses. Notably, we find a number of genes and pathways that have been previously associated with response and resistance are, in fact, differentially expressed in response to the drug in sensitive and resistant myeloma PCs.

We observed strikingly lower number of genes that changed significantly post-treatment in Ix-resistant HMCLs when compared with the Ix-sensitive HMCLs. A large number of these under-perturbed genes belonged to the PUP, suggesting a possible mechanism of PI resistance where this pathway is not sufficiently affected following PI treatment in relatively resistant HMCLs. For example, the gene PSMC6, the lack of which has been associated with cells having reduced chymotrypsin-like proteasome activity by PIs^12^. In our study, the upregulation of PSMC6 was more than twofold higher in the sensitive lines compared with Ix-resistant HMCLs. In addition to the PUP, the kinetic genes that were differentially responsive to PI treatment in Ix-sensitive HMCLs and patient-derived primary cells included NRF2-mediated oxidative stress response and oxidative stress response genes, that also corroborated with our current finding on kinetic GEP response in HMCLs reported here^13^. The NRF2-mediated oxidative stress response pathway has been shown earlier to control ER-stress induced apoptosis in myeloma in UPR^14^. Furthermore, among Ix-sensitive models, XBP1 inhibition and NFE2L2 activation were predicted as top upstream regulators based on the observed significantly regulated gene expression changes. Earlier studies have shown that XBP1 regulation is associated with significant inhibition of myeloma tumors which in turn is associated with chronic endocrine reticulum stress from UPR^15^. Thus, it was particularly interesting that XBP1 expression is differentially regulated in response to Ix. As described below, we found other genes whose expression has been associated with response and resistance are, in fact, differentially regulated by treatment with PIs.

The HERC1 gene belongs to the HERC family that has ubiquitin ligase activity dependent on the protein substrates, HERC proteins may act as a tumor suppressor or oncoprotein in specific cancer types. For example, HERC4 is believed to contribute to carcinogenesis of solid tumors such as lung cancer, but it suppresses the proliferation of myeloma cells^14^.

Among the genes uniquely expressed following Ix-treatment in resistant myeloma were RICTOR (activated), HNF4A, miR-16-5p (activated), MYCN (inhibited), and MYC (inhibited).

RICTOR is a scaffold protein that activates mTORC2 complex constituting mammalian Target of Rapamycin or mTOR, an intracellular serine/threonine kinase involved in intracellular cell survival, tumor growth, and drug resistance. Regulation of mTOR2 has been shown to influence myeloma angiogenesis^16^.

Major microRNA genes expressed uniquely in Ix-resistant patient cells included miR-16-5p (activated). An earlier study that used microarray profiling of tumor-derived exosomal RNA showed downegulation of miR-16-5p in circulating exosomes of patients resistant to PIs^17^. A number of studies have suggested that tumor-derived exosomal miRNA have a crucial role in the cross-talk between the tumor and its microenvironment and is also predictive of drug resistance in myeloma patients^17,18^.

One of the most notable findings of our study was that eIF2 signaling was the most significantly affected canonical pathway unique to the Ix-resistant cells. eIF2 signaling is involved in regulating ternary complex formation and protein synthesis^19^. Earlier studies have shown that inhibition of the 26 S proteasome results in a rapid decrease in the rate of protein synthesis owing to phosphorylating alpha subunit of the eukaryotic translation initiation factor 2 (eIF2α) by the heme-regulated inhibitor kinase (HRI)^20^. Further, dysregulation of the PERK-eIF2α pathway was involved in the efficiency of and sensitivity to target PIs^21–23^.

MYC genes are highly deregulated proto-oncogenes in human cancer. Aberrant MYC expression is frequently associated with cancer cell vulnerability and poor prognosis^24^. Several studies have therefore proposed the therapeutic targeting of aberrant MYC expression in hematological cancers including myeloma^24^. In our study, we found the MYC family genes were predicted to be inhibited based on the gene expression profile of the Ix-resistant cells. This may not be surprising, as we have previously demonstrated that the impact of myc expression on survival is very context dependent. High levels of myc expression in our previously reported transgenic mouse model in fact was associated with high cell death in the plasma cells expressing it^25^. Thus, the decrease in expression of myc pathway genes may well be associated with resistance to cell death.

IL-17A has been shown to influence myeloma cell proliferation and tumor growth through IL-17A receptor (IL-17RA)^4^ expressed on tumor cells^26^. Moreover, anti-IL-17A mAb could significantly inhibit myeloma cell-growth and survival both in vitro and in vivo^27^. Interestingly, in our IPA analysis, IL-17A signaling was the top canonical pathway for the 149 genes uniquely expressed in PI-resistant patient cells following treatment with PI.

IPA’s upstream analysis based on the genes found consistently downregulated with increase in dose (0 vs 20 nm vs 40 nm) predicted inhibition of the gene MITF as the top upstream regulator (*Z* score = −4.191; *P* = 1.08E-04). Earlier studies have shown that MITF is a direct target of miR-137, and miR-137-targeting of MITF regulates drug sensitivity in myeloma cells by reducing c-MET expression and suppressing AKT phosphorylation, accompanied by an increase in p53 expression^28^. Another study has found miR-137 directly targets and negatively regulates the protein expression of Enhancer of zeste 2 polycomb repressive complex 2 subunit (EZH2)—a histone methyltransferase that we have earlier shown to influence B-cell development, disease progression and treatment outcomes in myeloma^29–31^.

When the DE gene sets representing signatures of Ix were uploaded as separate data sets into the IPA/IPA software, the following revelations came forward. Although both the gene lists (Ix-sensitive and Ix-resistant myelomas) identified the PUP as the top canonical pathways, the overlap was around two times higher for the subgroups that respond to Ix treatment compared with the Ix-resistant subgroups. Furthermore, the UPR pathway was ranked higher among Ix-sensitive subgroup compared with the top canonical pathways observed in the subgroups that were relatively more resistant to Ix/PIs treatment. We also found, both in HMCLs and patient cells, that the treatment-induced increase in the involvement of the heat shock proteins and PUP was PI-dose dependent (not shown). HSPs play a crucial role in a number of biological processes involved in the maintenance of cellular protein homeostasis in a normal cells as wells as in cancer cells, including protein folding, cellular proliferation, differentiation, survival, metastasis, invasion, and angiogenesis^32–39^. In myeloma cells, HSPs play important role by activating the cytoprotective heat shock response during PI treatment with HSP27, HSP40, and HSP70 being highly unregulated following Btz treatment^39,40^. Btz has also been shown to induce time-dependent increased expression of HSP27, HSP70, and HSP90, whereas inducing apoptosis through proteasome inhibition in MM1S cells^39,40^. Upregulation of HSP90 have been observed in hematological malignancies including myeloma which is required for the stability and function of oncoproteins thereby supporting tumor development^41–43^. Our western blotting data also confirmed our transcriptomic analysis findings where Ix increased HSP40, HSP70b1, and HSP90α protein expression in Ix-sensitive HMCLs with little or no effect in Ix-resistant HMCLs. HMCLs representing intermediate PI response (IM-9, ARP-1, MOLP-8, MC CAR, U266, and RPMI-8226) showed variable kinetic PI-response levels.

Shaughnessy et al. performed Btz test dosing on a training set of 142 Total Therapy 3 A (MM-TT3A) patients and a validation set of 128 TT3B patients and identified a GEP80-postBz kinetic expression signature that included 80 highly survival-discriminatory genes^44^. When our kinetic Ix-response profiles in HMCLs and patients were compared with Shaughnessy’s GEP80-postBz, 10 genes (primarily involved in PUP) were found similar between all the subgroups (Fig. S5a), including PSMD4—the novel high-risk gene in myeloma patients^44^. Further, comparison of GEP80-postBz with our Ix-sensitive-only in vitro and ex vivo kinetic GEP signatures revealed 16 and 12 common (PUP) genes, respectively (Fig. S5b).

At last, for the most Ix-resistant HMCLs (LP1 and UTMC2), we added an additional Ix dose of 150 nm (10 times the test-dose of 15 nm) and performed gene expression analysis. We were particularly interested in DE genes that were consistently upregulated (fold-change > 1) or downregulated (fold-change < 1) among the three groups (no treatment vs 15 nm vs 150 nm).

Our results showed 331 genes were consistently downregulated (*p* < 0.05) between 0 vs 15 nm and 15 nm vs 150 nm treatments. In all, 493 genes that were consistently upregulated between 0 vs 15 nm (upregulated) and 15 nm vs 150 nm (upregulated) treatments. Thirty-two of these genes belong to the PUP (*P* = 1.12E-15).

Our results thus provide key insights into the difference between sensitive and resistant myeloma in terms of kinetic PI response. At present, we are using CRISPR-based gene knockdown of Hsp genes to explore novel mechanisms of PI resistance. This will eventually lead to future studies on the understanding of the biology governing the pharmacogenomics of differential PI response and the development of novel therapeutic approaches to circumvent the challenge.

## Supplementary information

Table S1

Supplementary Figures
