# Supplementary material for: In vitro and ex vivo gene expression profiling reveals differential kinetic response of HSPs and UPR genes is associated with PI resistance in multiple myeloma
Source: Blood Cancer J. 2020 Jul 28;10(7):78. doi: 10.1038/s41408-020-00344-9 (PMC7387444; doi:10.1038/s41408-020-00344-9)
Supplement: Supplementary file 1 — Table S1 [file 41408_2020_344_MOESM1_ESM.docx]

**Table S1** The top most highly regulated kinetic genes in a) PI-sensitive cell lines; b) PI-resistant cell lines; c) PI-sensitive patient cells; d) PI-sensitive patient cells. Cell lines were treated with test-dose of Ixazomib (a PI) and showed significantly de-regulated following drug exposure were identified comparing the Gene expression profiles of baseline vs treated.

a) PI-sensitive cell lines

|  | **pval** | **FOLD CHANGE** |
| --- | --- | --- |
| HMOX1 | 0.038 | 67.864 |
| HSPA1B | 0.039 | 41.511 |
| SQSTM1 | 0.027 | 19.443 |
| CLU | 0.025 | 15.154 |
| OSGIN1 | 0.028 | 14.260 |
| MLLT11 | 0.034 | 13.312 |
| FTL | 0.008 | 11.686 |
| RBM11 | 0.006 | 11.448 |
| GABARAPL1 | 0.003 | 9.598 |
| ROM1 | 0.001 | 9.269 |
| PPP1R15A | 0.037 | 8.336 |
| ATF3 | 0.016 | 8.208 |
| SRXN1 | 0.005 | 7.886 |
| B4GALNT1 | 0.027 | 7.751 |
| BAG3 | 0.047 | 7.224 |
| DNAJB1 | 0.024 | 6.924 |
| DYNC1I1 | 0.000 | 6.496 |
| GADD45A | 0.013 | 6.242 |
| MAPRE3 | 0.000 | 6.200 |
| UBC | 0.022 | 5.563 |
| MIR324 | 0.029 | 5.557 |
| TMSB15A | 0.026 | -5.550 |
| NUDT8 | 0.002 | -5.209 |
| MYB | 0.029 | -5.009 |
| STS | 0.025 | -4.995 |
| C16orf93 | 0.033 | 4.812 |
| HSP90AA1 | 0.017 | 4.810 |
| CCR10 | 0.045 | -4.780 |
| GPRC5D | 0.027 | -4.703 |
| C9orf25 | 0.024 | 4.630 |
| CYB5R1 | 0.001 | 4.605 |
| DGKG | 0.025 | 4.598 |
| SAT1 | 0.024 | 4.550 |
| SERPINB8 | 0.045 | 4.549 |
| VWA5A | 0.009 | 4.529 |
| BLVRB | 0.001 | 4.516 |
| MIDN | 0.011 | 4.495 |
| ITPKC | 0.029 | 4.487 |
| AIM2 | 0.000 | 4.309 |
| FKBP11 | 0.011 | -4.236 |
| GCLM | 0.000 | 4.213 |
| BRF2 | 0.001 | 4.210 |
| BHLHA15 | 0.002 | -4.159 |
| GLA | 0.004 | 4.136 |
| ANXA2 | 0.000 | 4.111 |
| PIR | 0.013 | 4.101 |
| SNORD10 | 0.008 | -4.064 |
| ABCB6 | 0.001 | 4.052 |
| ANXA2P1 | 0.007 | 4.033 |
| CXCR3 | 0.027 | -3.970 |

b) PI-resistant cell lines

|  | **pval** | **FOLD CHANGE** |
| --- | --- | --- |
| ANO10 | 0.010 | 1.112 |
| ANXA2P2 | 0.098 | 1.368 |
| BBS4 | 0.075 | 1.353 |
| BIRC6 | 0.099 | 1.162 |
| DYNC1I1 | 0.083 | 1.627 |
| ERCC6 | 0.042 | 1.236 |
| GSR | 0.096 | 1.256 |
| HERC1 | 0.061 | 1.220 |
| LRRC28 | 0.027 | 1.300 |
| NFAT5 | 0.079 | 1.292 |
| NMT1 | 0.094 | 1.150 |
| NSFL1C | 0.076 | 1.422 |
| PIR | 0.048 | 2.355 |
| PPIP5K1 | 0.025 | 1.213 |
| PSMA1 | 0.100 | 1.335 |
| PSMB3 | 0.091 | 1.308 |
| PSMB5 | 0.069 | 1.280 |
| PSMC4 | 0.098 | 1.544 |
| PSMC6 | 0.071 | 1.289 |
| PSMD2 | 0.050 | 1.482 |
| ROM1 | 0.095 | 1.729 |
| SNORA4 | 0.069 | -5.183 |
| SNORA62 | 0.058 | 5.473 |
| SNRPG | 0.093 | -1.772 |
| TMEM205 | 0.095 | 1.342 |
| TMEM97 | 0.064 | -1.104 |
| TNKS | 0.049 | 1.232 |
| VRK3 | 0.060 | 1.190 |
| ZYG11B | 0.078 | 1.251 |

c) PI-sensitive patient cells

|  | **pval** | **FOLD CHANGE** |
| --- | --- | --- |
| MIR1248 | 0.039 | 122.230 |
| RNY4 | 0.000 | 105.792 |
| HMGB1 | 0.000 | -61.593 |
| NDUFS4 | 0.025 | -37.017 |
| MLLT11 | 0.023 | 33.789 |
| CLU | 0.010 | 31.952 |
| RNU5B-1 | 0.000 | 24.720 |
| FICD | 0.048 | -23.658 |
| RNU5A-1 | 0.000 | 23.434 |
| NIFK | 0.000 | -23.136 |
| SNORA18 | 0.015 | 22.577 |
| SRXN1 | 0.006 | 21.216 |
| SNORA81 | 0.000 | 21.017 |
| SNRPD1 | 0.000 | -20.494 |
| PRDX4 | 0.029 | -18.592 |
| EIF4EBP1 | 0.013 | -18.467 |
| SQSTM1 | 0.000 | 17.985 |
| HBD | 0.012 | -16.567 |
| HINT2 | 0.008 | -16.181 |
| DLST | 0.003 | -15.587 |
| IDH2 | 0.046 | -15.436 |
| NME1 | 0.009 | -15.423 |
| PADI4 | 0.021 | 14.481 |
| CEP63 | 0.001 | -14.234 |
| GMPS | 0.014 | -13.807 |
| CCDC86 | 0.040 | -13.662 |
| TMEM258 | 0.015 | -13.635 |
| SNORD116-26 | 0.015 | 13.500 |
| LYAR | 0.009 | -13.374 |
| SNORA23 | 0.006 | 13.197 |
| FYB | 0.039 | 12.997 |
| STK25 | 0.000 | -12.967 |
| PLEKHJ1 | 0.014 | -12.893 |
| RRP36 | 0.000 | -12.837 |
| CCDC50 | 0.035 | -12.765 |
| NOP16 | 0.001 | -12.318 |
| BRF2 | 0.038 | 11.954 |
| RNY1 | 0.003 | 11.821 |
| ZFAND2A | 0.044 | 11.721 |
| C10orf35 | 0.041 | 11.698 |
| ZNF706 | 0.000 | -11.622 |
| POU2F2 | 0.042 | -11.550 |
| RHOH | 0.015 | -11.496 |
| MRPL47 | 0.000 | -11.485 |
| DNAJB1 | 0.046 | 11.372 |
| ALOX12P2 | 0.044 | 10.749 |
| RNY3 | 0.015 | 10.526 |
| CHCHD7 | 0.000 | -10.498 |
| APOBEC3G | 0.024 | -10.187 |
| SP140 | 0.036 | -10.012 |

d) PI-sensitive patient cells

|  | **pval** | **FOLD CHANGE** |
| --- | --- | --- |
| B4GALNT1 | 0.036 | 19.648 |
| TIMM10 | 0.024 | -16.544 |
| ALG5 | 0.007 | -14.925 |
| MRPS12 | 0.025 | -12.351 |
| RGS3 | 0.014 | -12.213 |
| COA7 | 0.001 | -11.371 |
| LINC00493 | 0.006 | -11.337 |
| CYP51A1 | 0.009 | -10.811 |
| NUGGC | 0.004 | -10.515 |
| UBE2V2 | 0.021 | -10.403 |
| C18orf32 | 0.013 | -10.256 |
| PARP2 | 0.004 | -10.215 |
| FBXO4 | 0.047 | -10.126 |
| POLR3K | 0.030 | -9.872 |
| TRAPPC2L | 0.011 | -9.803 |
| SEC61G | 0.004 | -9.615 |
| ARV1 | 0.016 | -9.518 |
| GPX7 | 0.001 | -9.276 |
| FAM118B | 0.013 | -9.218 |
| FAM188A | 0.002 | -9.203 |
| MRPS26 | 0.022 | -9.106 |
| BIK | 0.013 | -9.072 |
| AGA | 0.006 | -9.069 |
| RPP25L | 0.005 | -9.003 |
| NDUFS7 | 0.008 | -8.849 |
| C10orf2 | 0.003 | -8.797 |
| UBXN2A | 0.001 | -8.752 |
| RSC1A1 | 0.013 | -8.599 |
| NKAP | 0.013 | -8.351 |
| ISOC2 | 0.016 | -8.268 |
| POP5 | 0.011 | -8.175 |
| TMED8 | 0.020 | -8.095 |
| ORMDL2 | 0.008 | -8.009 |
| GSKIP | 0.033 | -7.980 |
| PSMG4 | 0.005 | -7.948 |
| NOP16 | 0.022 | -7.708 |
| PRCP | 0.000 | -7.672 |
| C10orf54 | 0.016 | -7.650 |
| RABGEF1 | 0.007 | -7.545 |
| SRM | 0.019 | -7.541 |
| TEN1 | 0.004 | -7.509 |
| TXNL4A | 0.005 | -7.468 |
| CSTF3 | 0.009 | -7.446 |
| PRKAR2A | 0.021 | -7.429 |
| GSPT2 | 0.005 | -7.423 |
| RPL9 | 0.012 | -7.319 |
| PIGC | 0.004 | -7.267 |
| MIF4GD | 0.001 | -7.181 |
| SRA1 | 0.003 | -7.159 |
| RPL39 | 0.009 | -7.097 |
