# Supplementary material for: In vitro and ex vivo gene expression profiling reveals differential kinetic response of HSPs and UPR genes is associated with PI resistance in multiple myeloma
Source: Blood Cancer J. 2020 Jul 28;10(7):78. doi: 10.1038/s41408-020-00344-9 (PMC7387444; doi:10.1038/s41408-020-00344-9)
Supplement: Supplementary file 2 — Supplementary Figures [file 41408_2020_344_MOESM2_ESM.pptx]

## Slide 1
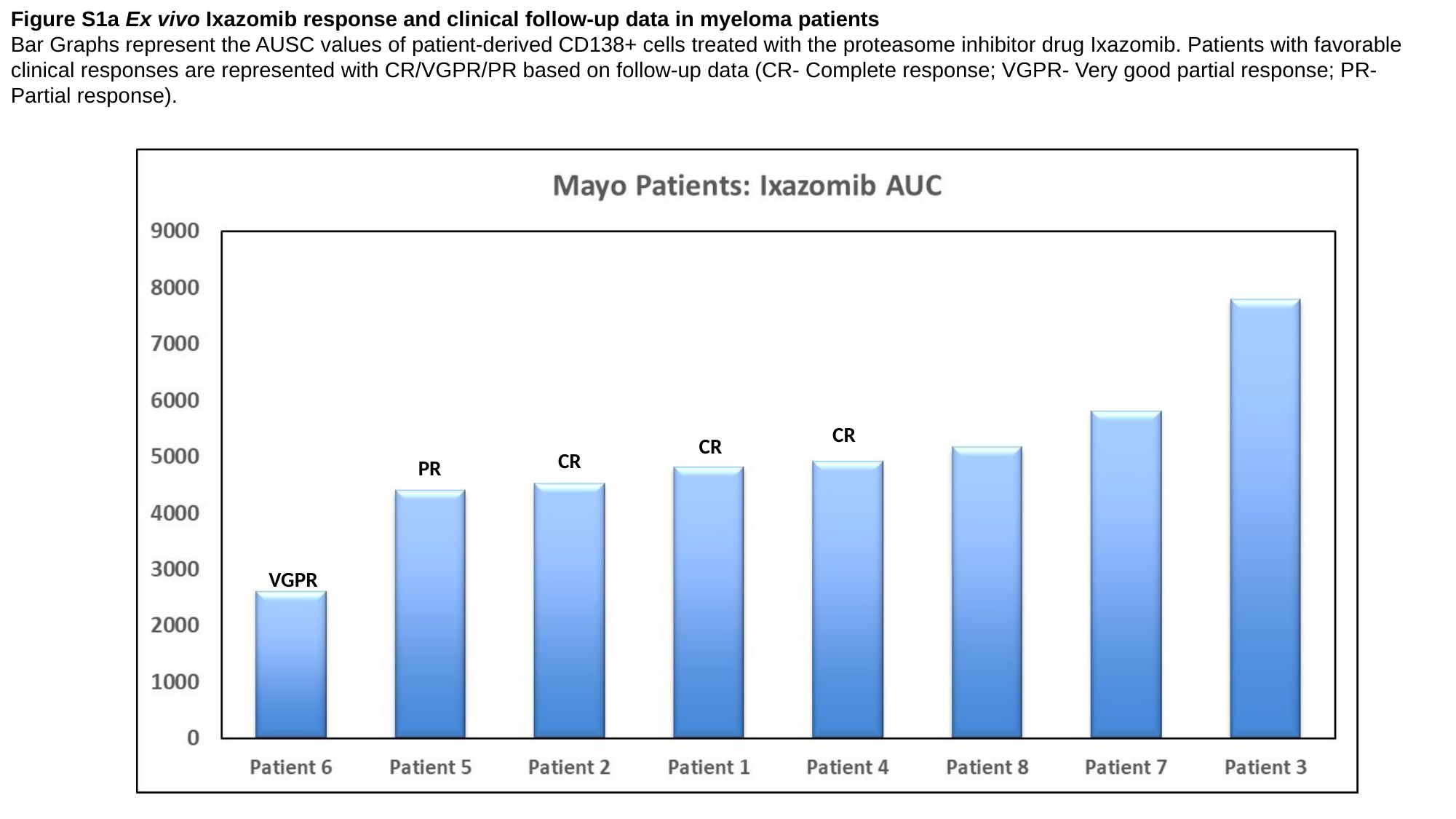

Figure S1a Ex vivo Ixazomib response and clinical follow-up data in myeloma patients
Bar Graphs represent the AUSC values of patient-derived CD138+ cells treated with the proteasome inhibitor drug Ixazomib. Patients with favorable clinical responses are represented with CR/VGPR/PR based on follow-up data (CR- Complete response; VGPR- Very good partial response; PR- Partial response).
CR
CR
CR
PR
VGPR

## Slide 2
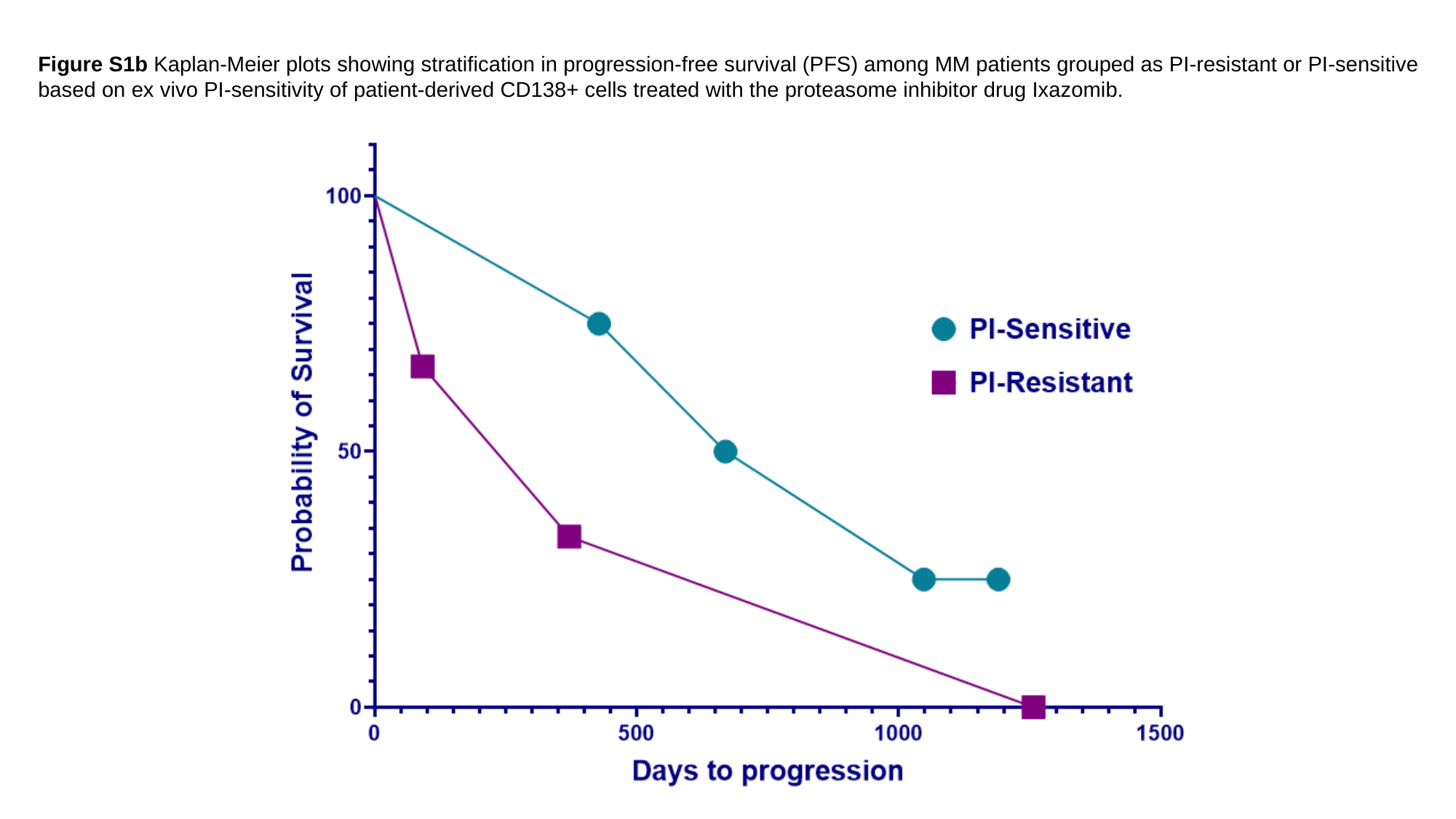

Figure S1b Kaplan-Meier plots showing stratification in progression-free survival (PFS) among MM patients grouped as PI-resistant or PI-sensitive based on ex vivo PI-sensitivity of patient-derived CD138+ cells treated with the proteasome inhibitor drug Ixazomib.

## Slide 3
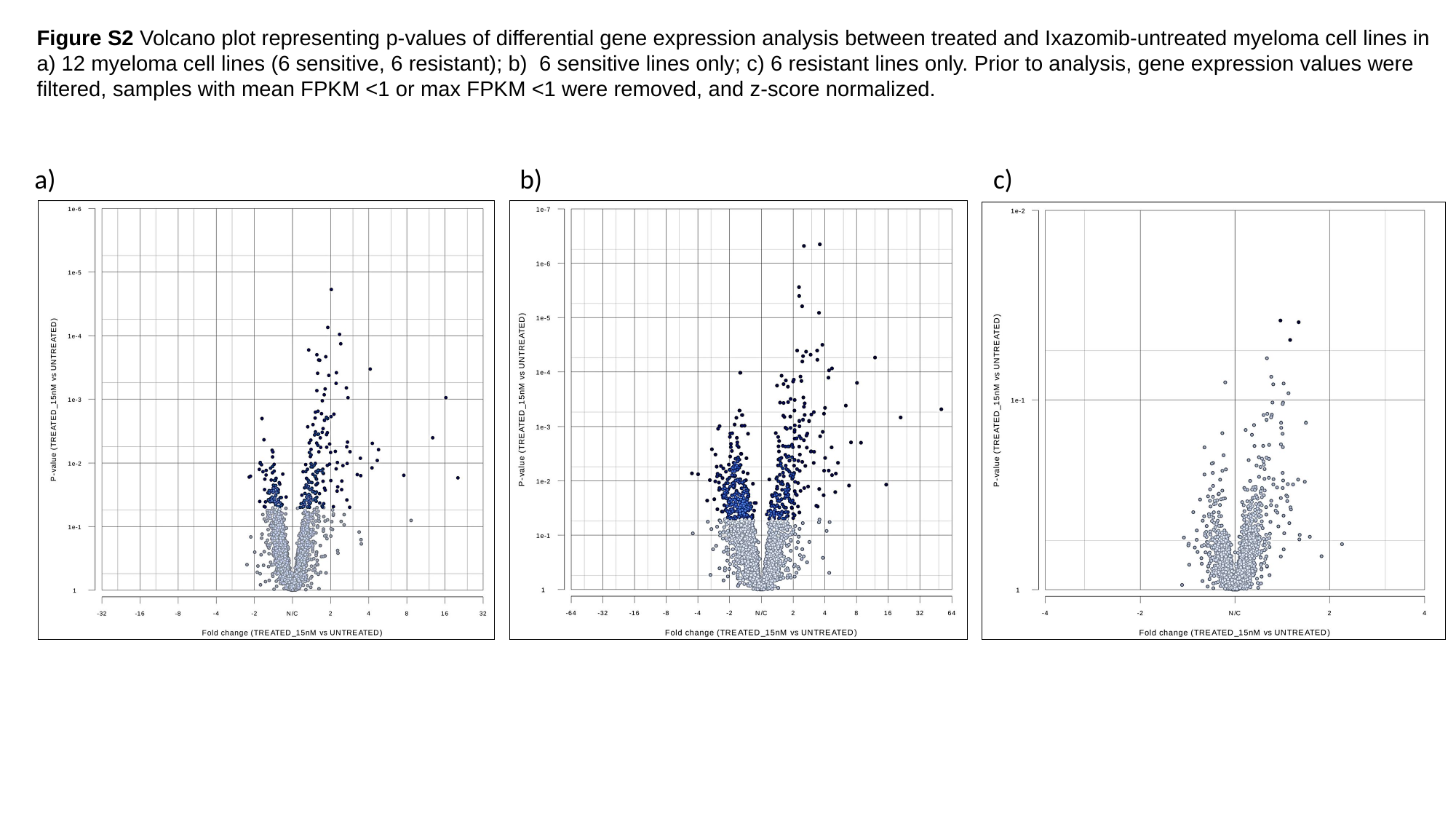

Figure S2 Volcano plot representing p-values of differential gene expression analysis between treated and Ixazomib-untreated myeloma cell lines in a) 12 myeloma cell lines (6 sensitive, 6 resistant); b) 6 sensitive lines only; c) 6 resistant lines only. Prior to analysis, gene expression values were filtered, samples with mean FPKM <1 or max FPKM <1 were removed, and z-score normalized.
a)
b)
c)

## Slide 4
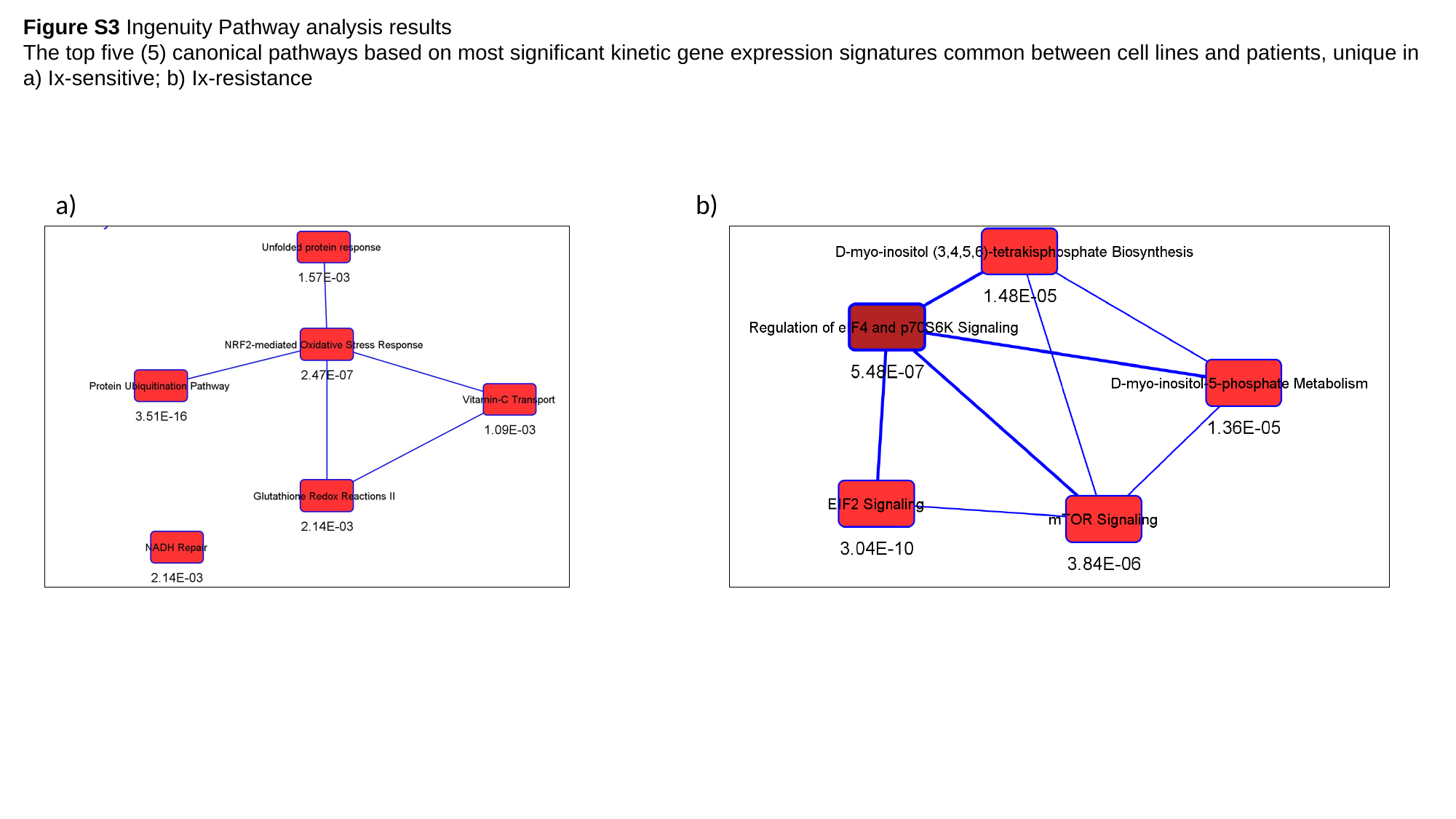

Figure S3 Ingenuity Pathway analysis results
The top five (5) canonical pathways based on most significant kinetic gene expression signatures common between cell lines and patients, unique in
a) Ix-sensitive; b) Ix-resistance
a)
b)

## Slide 5
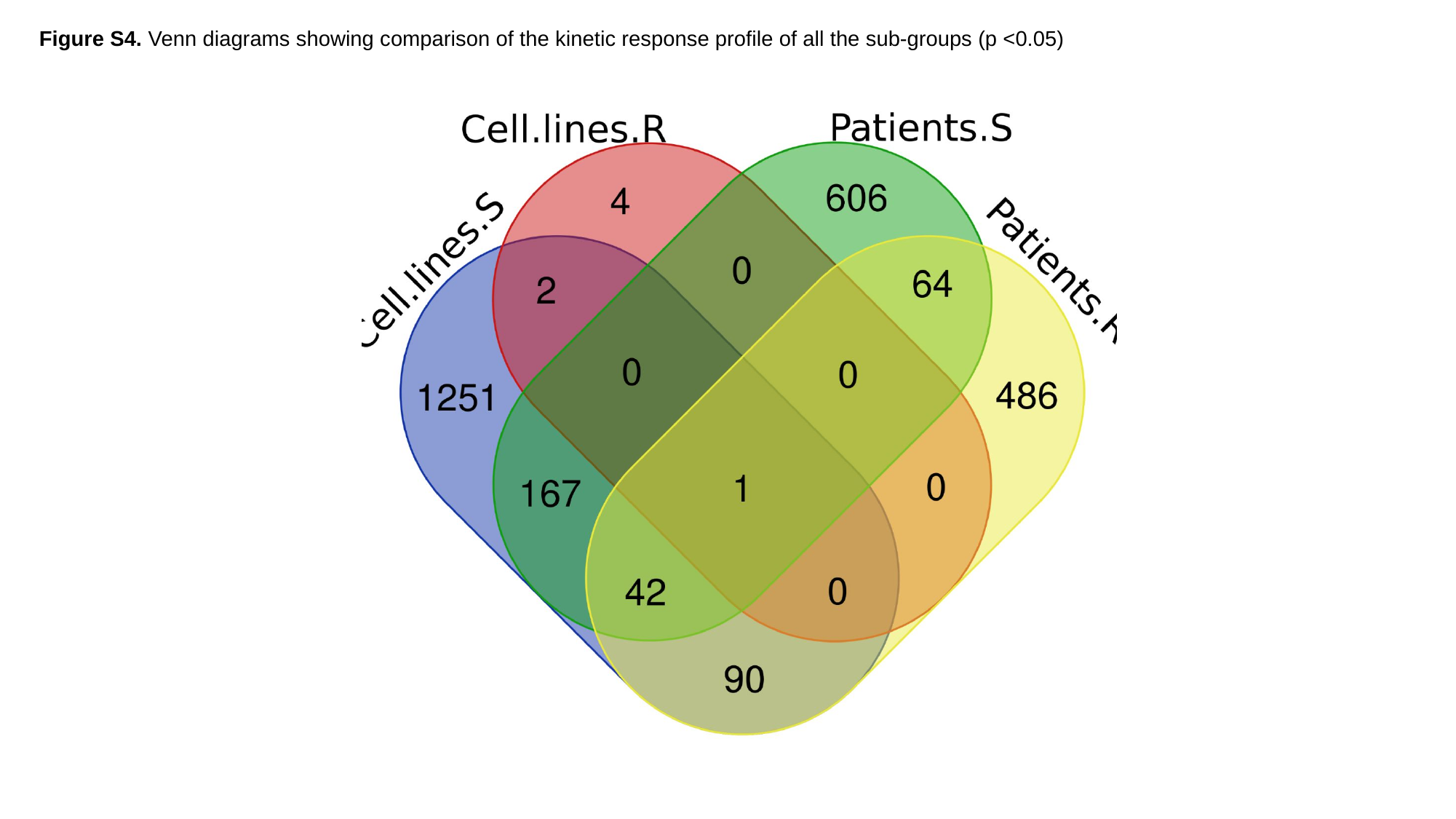

Figure S4. Venn diagrams showing comparison of the kinetic response profile of all the sub-groups (p <0.05)

## Slide 6
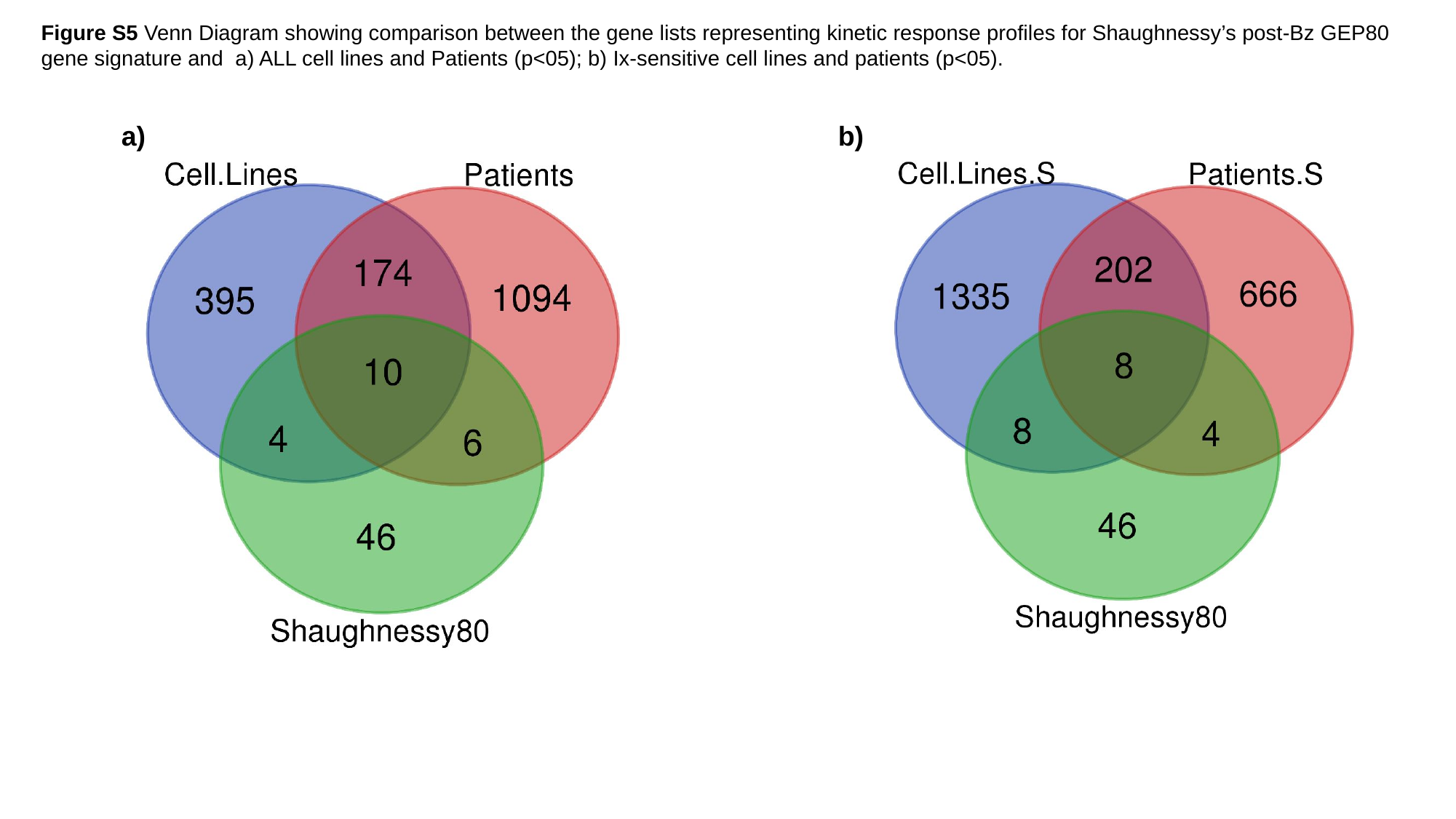

Figure S5 Venn Diagram showing comparison between the gene lists representing kinetic response profiles for Shaughnessy’s post-Bz GEP80 gene signature and a) ALL cell lines and Patients (p<05); b) Ix-sensitive cell lines and patients (p<05).
a)
b)
